# Design-Stage Feasibility Study of an Integrated 3D-Printed Nerve Conduit and Portable Monitoring System

**DOI:** 10.3390/ma19194029

**Published:** 2026-09-22

**Authors:** Leonard Gabriel Mitu, Miriam Maxim, Ileana Pantea, Angela Repanovici

**Affiliations:** Faculty of Product Design and Environment, Transilvania University of Brasov, 500036 Brasov, Romania; leonard.mitu@unitbv.ro (L.G.M.); miriam.maxim@student.unitbv.ro (M.M.)

**Keywords:** 3D printing, PLA, microchannel conduit, ESP32, EMG monitoring, finite element analysis, additive manufacturing

## Abstract

(1) Background: Peripheral nerve repair requires structural guidance and reliable functional assessment, yet current commercial conduits lack internal microarchitecture and do not integrate real-time monitoring capabilities. This study presents an engineering-focused feasibility assessment of a dual system combining a microstructured 3D-printed nerve guidance conduit with a portable neuromuscular monitoring device. (2) Methods: Two conduit variants were digitally designed based on median nerve anatomical dimensions and fabricated using FDM (PLA) for large-scale prototypes and SLA for high-resolution miniaturized models. Structural behavior was evaluated through simplified finite element analysis (FEA) under physiological pressure ranges (1000–5000 Pa). A portable monitoring system incorporating surface EMG electrodes, an AD620 instrumentation amplifier, and an ESP32 microcontroller was assembled and tested non-invasively on a healthy adult volunteer to verify signal acquisition functionality. Results: Both conduit designs were successfully fabricated with accurate reproduction of internal microchannels. FEA indicated negligible deformation (1.41 × 10^−11^–1.69 × 10^−10^ mm) and low stress values (0.0129–0.155 N/m^2^), confirming structural stability under the simplified loading model. The monitoring system recorded stable EMG signals (3200–3500 ADC units), demonstrating correct operation of the acquisition chain during controlled stimulation. (3) Conclusions: This work provides a design-stage engineering feasibility demonstration of an integrated platform combining a microstructured 3D-printed conduit with a portable neuromuscular monitoring device. The study does not include biological validation; prototypes were evaluated solely for geometric and mechanical fidelity; and the monitoring system was tested only for functional signal acquisition. Future work will address biocompatibility, in vitro assays, and in vivo evaluation.

## 1. Introduction

Peripheral nerve injuries require structural support and precise functional assessment to enable effective repair strategies. Commercial nerve guidance conduits provide a protected pathway for bridging short-segment defects, yet they lack internal microarchitecture capable of guiding multiple fascicular pathways and cannot be customized to patient-specific anatomy [1,2]. Additive manufacturing offers a practical solution to these limitations, enabling rapid prototyping and fabrication of conduits with controlled internal geometry, including microchannels inspired by the fascicular organization of the median nerve [3,4,5].

In parallel, functional monitoring remains essential for evaluating neuromuscular activity during recovery or experimental testing. Conventional neurophysiological systems provide accurate measurements but are expensive, non-portable, and require specialized personnel. Portable EMG devices exist, but they are typically optimized for high-amplitude muscle activity rather than low-amplitude signals relevant to early nerve activity or controlled stimulation scenarios. This creates a technological gap for compact, low-cost systems capable of real-time neuromuscular signal acquisition in preclinical or engineering contexts.

The present study addresses these two engineering needs by developing:

(1) A 3D-printed nerve guidance conduit incorporating internal microchannels;
(2) A portable neuromuscular monitoring device based on surface EMG acquisition.

This work is explicitly positioned as a design-stage engineering feasibility study. The conduits were fabricated solely for geometric and mechanical validation, and the monitoring system was evaluated only to confirm functional signal acquisition. No biological testing or regeneration assessment was performed.

## 2. Materials and Methods

### 2.1. Design and Fabrication of the 3D Nerve Guidance Conduit

#### 2.1.1. Material Selection

Polylactic acid (PLA) was selected as the fabrication material based on its established biocompatibility, controlled biodegradability, and suitability for additive manufacturing. Under biological conditions (37 °C, neutral pH), PLA undergoes hydrolytic degradation through hydrolysis of ester bonds, producing lactic acid, a biocompatible metabolite naturally processed by the organism [6]. The glass transition temperature of PLA (Tg ≈ 55–60 °C) exceeds the physiological operating temperature by a sufficient margin, ensuring structural stability without deformation in vivo. For SLA fabrication, a standard photopolymer resin compatible with the Anycubic Photon Mono M7 Max printer (Shenzhen Anycubic Technology Co., Ltd., Shenzhen, China) was used to achieve higher-resolution miniaturized prototypes; this resin was used exclusively for geometric validation and does not possess biocompatibility properties. The printed conduits were produced exclusively for geometric and mechanical evaluation. As stated, the SLA resin ‘does not possess biocompatibility properties,’ and PLA prototypes were not subjected to cytocompatibility, degradation, or Schwann cell adhesion assays [6]. No biological validation was performed at this stage.

#### 2.1.2. Digital Design

Two conduit variants were designed using CATIA P3 V5-6R2022 CAD software, based on published anatomical data for the median nerve. According to a systematic review encompassing 73 clinical studies, the mean cross-sectional area (CSA) of the median nerve at the carpal tunnel is approximately 8.60 mm^2^ [7]. The inner conduit diameter was derived using the relation d = $\sqrt{\left(A/\pi \right)}$ , yielding approximately 3.31 mm. A safety margin of 0.2 mm was applied to avoid compression of the nerve, resulting in a final inner diameter of 3.5 mm and an outer diameter of 4 mm.

Both conduit variants share a total length of 10 mm, comprising an 8 mm active segment containing internal microchannels, and 1 mm solid sections at the proximal and distal ends, each featuring 0.4 mm suture holes for surgical fixation. The variants differ in their internal microchannel architecture: Conduit A incorporates 6 symmetrically arranged fascicular channels, while Conduit B incorporates 4 irregularly arranged channels. Each microchannel includes a central channel reserved for potential integration of neurotrophic factors (e.g., NGF, bFGF) in future iterations [8,9]. 

#### 2.1.3. Additive Manufacturing

Two complementary printing technologies were employed, each targeting a different prototype scale. FDM using PLA filament on a Prusa MK4 printer (Prusa Research, Prague, Czech Republic) was used for large-scale structural prototypes, enabling rapid geometric iteration and visual verification of the internal architecture. SLA using photopolymer resin on an Anycubic Photon Mono M7 Max printer (Shenzhen Anycubic Technology Co., Ltd., Shenzhen, China) was used for high-resolution miniaturized models, enabling finer surface detail and closer replication of microchannel geometry at the target implantation scale. Both prototype sets were fabricated solely for design validation purposes.

#### 2.1.4. Mechanical Validation

Finite element analysis (FEA) was performed in CATIA P3 V5-6R2022 (Generative Structural Analysis module) to evaluate the conduit’s structural behavior under simulated biological pressure conditions. PLA material properties were defined in the software material library according to published data: Young’s modulus 3.5 × 10^9^ N/m^2^, Poisson’s ratio 0.36, density 1240 kg/m^3^, yield strength 60 × 10^6^ N/m^2^, and thermal expansion coefficient 6.8 × 10^−5^ K^−1^ [10]. The mesh was generated using the OCTREE algorithm with an element size of 0.5 mm and a maximum geometric sag of 0.1 mm. Fixed constraints (clamp) were applied to the suture hole surfaces to simulate rigid surgical anchoring. Distributed pressure loads of 1000 Pa and 5000 Pa were applied to the outer conduit surface, corresponding to the physiological compressive pressure range reported for carpal tunnel soft tissue [11].

### 2.2. Development of the Portable Monitoring System

#### 2.2.1. System Architecture

The monitoring system was designed to capture, filter, amplify, and visualize low-amplitude neuromuscular signals generated in response to external nerve stimulation. The system targets low-level signals characteristic of peripheral nerve activity and surface-recorded responses. The signal acquisition chain follows five sequential functional stages: surface detection → passive filtering → differential amplification → digital acquisition → real-time display.

#### 2.2.2. Hardware Components

The EMG H124SG surface electrode module (Sigmanortec S.R.L., Târgu Jiu, Romania) captures raw analog neuromuscular signals generated in response to median nerve activity (1–100 µV), powered at ±3.5–9 VDC. The signal passes through a passive RC low-pass filter (R = 1 kΩ, C = 0.22 µF), designed to attenuate high-frequency noise and power-line interference while preserving the clinically relevant EMG frequency band, with cutoff frequency defined by fc = 1/(2πRC). The filtered signal is transmitted to an AD620 instrumentation amplifier modul (Analog Devices, Inc., Norwood, MA, USA)e for differential amplification, with gain adjustable via external potentiometer W104 (100 kΩ) according to G = 1 + 49.4 kΩ/R_G, and a second potentiometer W103 (5 kΩ) enabling fine adjustment of the reference voltage (Vref) to center the amplified signal. An ESP32-WROOM-32S microcontroller (Sigmanortec S.R.L., Târgu Jiu, Romania) performs analog-to-digital conversion via GPIO34, signal processing, and optional wireless data transmission over Wi-Fi. A TFT LCD ST7735 display (Optimus Digital S.R.L., Bucharest, Romania) (1.8”, 3.3 VDC) provides real-time graphical visualization of the acquired waveform. All components were integrated onto a custom 7 × 9 cm PCB, housed in a protective industrial enclosure.

#### 2.2.3. Software Implementation

Firmware was developed in Arduino IDE 2.3.7 for the ESP32 platform. The software performs analog signal acquisition via UART at a sampling rate sufficient to capture SNAP and CMAP dynamics, real-time graphical rendering on the TFT display, and Vref-based calibration for signal baseline centering. The system supports both USB-powered and autonomous battery-powered operation via a 3.7 V Li-ion 18,650 cell, with wireless data streaming to a local hotspot for cable-free monitoring.

#### 2.2.4. Ethical Considerations

The functional test of the monitoring system involved a single healthy adult volunteer (author M.L.G.). The procedure was entirely non-invasive, using only surface EMG electrodes, and was performed with informed consent. According to institutional guidelines for non-invasive engineering device testing, no formal ethics committee approval was required.

## 3. Results

### 3.1. Fabrication of the Nerve Guidance Conduit Prototypes

After finalizing the design in CATIA V5, both conduit models were exported in STL format, checked for geometric errors in PrusaSlicer 2.9.0, and prepared for fabrication using technology-specific parameters. The internal microchannel architecture of both conduits was inspired by the natural guidance structures formed during peripheral nerve regeneration: the Bands of Büngner, cellular tracks created by Schwann cells that direct regenerating axons toward their distal targets [12,13]. Conduit B, with its four irregularly arranged channels, more closely approximates the non-uniform spatial distribution observed in native Büngner band formation, while Conduit A adopts a symmetric six-channel arrangement for comparative structural evaluation [12]. The CAD projections of Conduit A and Conduit B are presented in (Figure 1 and Figure 2). Large-scale FDM prototypes (outer diameter 40 mm, height 100 mm) were successfully fabricated from PLA filament on the Prusa MK4 printer. These models allowed visual and tactile verification of the overall conduit geometry, wall thickness, and proximal/distal end sections. Miniaturized SLA prototypes were subsequently produced on the Anycubic Photon Mono M7 Max: one at a reduced verification scale (8 mm diameter) and one at the target implantation dimensions (4 mm outer diameter). The SLA process achieved finer surface resolution, enabling clearer visualization of the internal fascicular microchannel arrangement. All prototypes successfully reproduced the intended architecture: longitudinal microchannels, proximal and distal suture hole sections, and the central channel reserved for neurotrophic factor integration (Figure 3).

To verify that the longitudinal microchannels were fully open and contiguous along the entire 10 mm length, the SLA miniaturized conduits were sectioned and inspected under magnification. All channels were confirmed to be patent, with no occlusions or collapsed regions. This step ensures that regenerating axons would not encounter blocked pathways, addressing a critical requirement for nerve guidance conduits.

The internal microchannel architecture was inspired by the longitudinal organization of fascicular pathways described in anatomical studies [12]. The design intent was to reproduce parallel microchannels for structural guidance, without implying biological functionality.

### 3.2. Finite Element Analysis of the Conduit

The FEA simulation results confirmed the structural integrity of the conduit under both pressure conditions. Distributed pressure loads of 1000 Pa and 5000 Pa were applied to the outer conduit surface (Figure 4). The displacement field maps showed maximum nodal displacement of 1.41 × 10^−11^ mm under 1000 Pa and 1.69 × 10^−10^ mm under 5000 Pa (Figure 5). These values are negligible in the context of any clinically relevant deformation. Von Mises stress analysis yielded maximum values of 0.0129 N/m^2^ at 1000 Pa and 0.155 N/m^2^ at 5000 Pa, both several orders of magnitude below PLA’s yield strength of 60 × 10^6^ N/m^2^ (Figure 6). The color-mapped stress distributions confirmed that peak stress concentrations occur near the fixed suture hole constraints, while the active microchannel segment remains mechanically unaffected under all tested loads. These results validate the conduit’s mechanical stability under physiological pressure conditions expected in the soft tissue environment during peripheral nerve regeneration. The extremely small displacements observed (e.g., ‘1.41 × 10^−11^ mm’) result from the simplified pressure-only loading model. This simulation is intended as an initial structural feasibility check rather than a full physiological representation of soft tissue interactions. The mesh was refined using an OCTREE algorithm (element size 0.5 mm, sag 0.1 mm). Boundary conditions consisted of clamped constraints applied to the suture hole surfaces. 

### 3.3. Assembly and Testing of the Monitoring System

Prior to PCB soldering, all components were individually verified on a breadboard. The RC filter resistor was measured with a digital multimeter, confirming the target cutoff frequency of fc ≈ 723.4 Hz. AD620 potentiometers were configured as follows: W104 set to R_G = 1 kΩ, yielding G = 1 + 49,400/1000 ≈ 50.4, sufficient to amplify a 50 µV input to approximately 2.5 mV, within the ESP32 ADC input range, and W103 set to 5 kΩ for optimal Vref offset centering. Total estimated current consumption was 231.5 mA, yielding an autonomy of approximately 5 h on the 1200 mAh Li-ion cell. Following successful breadboard validation, components were soldered onto the PCB and the TFT display was flush-mounted through the enclosure lid, providing direct user access to the real-time signal interface.

### 3.4. Neuromuscular Signal Acquisition

Preliminary functional testing was conducted using a TENS device set to acupuncture mode, level 5/20 (stimulation frequency ≈ 25 Hz). Stimulation electrodes were placed over the median nerve trajectory in the forearm to induce controlled neuromuscular activation. Recording electrodes were positioned as follows: reference electrode (GND) at the elbow, electrode A over the carpal tunnel along the median nerve axis, and electrode B at the base of the index–middle finger interspace (Figure 7). The recorded EMG signal consistently ranged between 3200 and 3500 digital units on the ESP32*’*s 12-bit ADC scale (0–4095), corresponding to an estimated voltage of 2.5–3.0 V. This test confirms only the correct operation of the acquisition chain. As stated in the manuscript, ‘the monitoring system was evaluated on a healthy subject,’ and therefore the results do not assess nerve regeneration. This range falls within the high-activity classification threshold (>3000 units) of the implemented interpretation algorithm, displayed as *“*Nerve active*/*Status: HEALTHY*”* in green on the TFT interface. No signal dropout or latency anomalies were observed during testing. These results provide preliminary validation of the system*’*s signal acquisition chain and confirm correct stimulation-recording setup, while also offering an initial indication of functional median nerve conduction in the tested subject.

The TFT LCD display provides real-time visualization of the acquired EMG signal, expressed as a digital value between 0 and 4095, corresponding to an analog voltage range of 0–3.3 V, consistent with the 12-bit resolution of the ESP32 ADC (2^12^ = 4096 levels). Based on the acquired value, the system displays a simplified neuromuscular activity classification using a three-tier color-coded scheme: values above 3000 trigger the message “Nerve active/Status: HEALTHY” in green, indicating strong neuromuscular activity and normal nerve function; values between 1500 and 3000 display “Nerve active/Status: WEAK” in yellow, suggesting reduced or incomplete neuromuscular response; and values below 1500 display “No signal/Check connections” in red, indicating either absence of neural response or a technical issue such as electrode disconnection or external interference.

The test on a single healthy subject was performed solely to verify the correct operation of the acquisition chain. This procedure does not constitute a physiological or clinical evaluation of nerve regeneration.

## 4. Discussion

This study presents an integrated engineering platform consisting of a microstructured 3D-printed conduit and a portable neuromuscular monitoring device. The fabrication results demonstrate that additive manufacturing can reliably reproduce internal microchannel architectures at both large-scale and miniaturized dimensions. SLA printing, in particular, enabled high-resolution replication of the intended geometry, supporting its suitability for future preclinical prototype development.

The structural evaluation performed through finite element analysis confirmed that the conduit remains mechanically stable under the simplified pressure conditions applied. The extremely small displacement values obtained (10^−11^–10^−11^ mm) reflect the limitations of the pressure-only loading model and the absence of soft tissue interactions. Therefore, the FEA results should be interpreted strictly as an initial structural feasibility check rather than a physiological simulation. Future simulations will incorporate more realistic boundary conditions, anisotropic material behavior, and soft tissue contact models.

The portable monitoring system successfully acquired stable EMG signals during controlled stimulation, demonstrating correct operation of the amplification, filtering, and acquisition chain. The test performed on a single healthy volunteer served exclusively to validate system functionality and does not represent a clinical or physiological evaluation. Future work will expand testing to additional subjects, refine signal processing algorithms, and explore integration with wireless data logging.

Overall, this study provides a design-stage engineering feasibility demonstration. The work is intentionally limited to geometric, mechanical, and functional validation. No biological outcomes, regeneration performance, or clinical recovery assessments were included. These aspects represent essential next steps, together with biocompatibility testing, in vitro assays, and in vivo evaluation.

### 4.1. Evaluation of the 3D-Printed Conduit in the Context of Existing Alternatives

Commercial nerve guidance conduits such as Neurolac, Reaxon, Neuroflex, NeuroMatrix, and NeuraGen have demonstrated clinical utility for bridging short-to-moderate nerve gaps [13]. However, these devices share fundamental limitations: they are manufactured in standardized geometries, lack patient-specific customization, and provide only a smooth internal lumen without fascicular microarchitecture [14,15]. The absence of internal guidance structures is clinically relevant, as unguided axonal sprouting increases the risk of misrouting, neuroma formation, and incomplete functional recovery, particularly in mixed nerves such as the median nerve.

The conduits developed in this study directly address these limitations by incorporating internal microchannels inspired by the natural Bands of Büngner, which play a critical role in directing regenerating axons [3,4]. Conduit A provides a symmetric six-channel configuration, while Conduit B offers a more irregular four-channel architecture that more closely resembles the heterogeneous fascicular distribution observed in vivo. Similar microstructured conduits have been shown to improve axonal alignment, reduce dispersion, and enhance functional recovery in preclinical models [15,16].

PLA was selected as a pragmatic material for this proof-of-concept stage due to its biocompatibility, controlled biodegradation, and compatibility with both FDM and SLA printing [14]. Although PLA lacks the intrinsic bioactivity of materials such as chitosan or collagen, its predictable degradation profile aligns with the typical regeneration timeline of the median nerve. Future iterations may incorporate composite materials or surface functionalization strategies to enhance Schwann cell adhesion and neurotrophic signaling, as demonstrated in recent studies using PLGA–collagen blends and chitosan-based conduits [15].

Finite element analysis confirmed that the conduit maintains structural integrity under physiological compressive pressures typical of the carpal tunnel. The extremely low displacement and stress values—several orders of magnitude below the PLA yield strength—indicate that the conduit is mechanically robust and unlikely to deform during implantation or early regeneration. These findings are consistent with previous studies demonstrating the suitability of PLA-based conduits for peripheral nerve repair [14,16]. This study should be interpreted as an engineering proof-of-concept. As stated throughout the manuscript, ‘the conduits were fabricated only for geometric and mechanical validation’ and ‘no biological testing was performed.’ The monitoring system was evaluated only on a healthy subject, confirming functional signal acquisition but not regenerative assessment. Future work will incorporate biocompatible materials, in vitro assays, in vivo implantation, and electrophysiological evaluation in nerve injury models.

We acknowledge that PLA and standard SLA resins are relatively rigid materials and do not provide the flexibility typically recommended for peripheral nerve implants. As stated in the manuscript, the conduits were fabricated ‘only for geometric and mechanical validation,’ and were not intended as final implantable devices. Future iterations will employ flexible biocompatible polymers such as PCL, TPU-based medical elastomers, or photocurable elastomeric resins, which better match the compliance of native nerve tissue.

### 4.2. Performance of the Portable Monitoring System

Conventional clinical electrodiagnostic systems provide high diagnostic accuracy but are limited by their cost, size, and requirement for specialized operators [17]. As a result, they are unsuitable for continuous monitoring or for use in low-resource research environments. Existing portable EMG devices offer improved accessibility but are typically optimized for high-amplitude muscle activity rather than the low-amplitude signals characteristic of early nerve regeneration [18].

The monitoring system developed in this study occupies an underserved design space by prioritizing accessibility, portability, and integration with the conduit prototype. The successful acquisition of stable EMG signals in the 3200–3500 ADC unit range demonstrates the validity of the signal acquisition chain, including the RC filtering stage, AD620 amplification, and ESP32-based digital processing. The three-tier classification interface provides intuitive, real-time feedback without requiring specialized electrophysiological training, and the system’s wireless capability enables remote monitoring during experimental sessions.

Although the system does not aim to replace clinical-grade NCS equipment, it provides a practical and scalable tool for preclinical research, enabling frequent assessments of neuromuscular activity at a fraction of the cost of conventional systems. Similar low-cost EMG platforms have been proposed in recent years, but none have been integrated with a structural nerve repair device, highlighting the novelty of the present approach [17]. 

Although the monitoring system currently provides a simplified three-level classification (‘HEALTHY’, ‘WEAK’, ‘NO SIGNAL’), this type of readout could support preclinical and low-resource clinical environments by enabling rapid screening of nerve activity without specialized equipment. Such a tool could help identify early reinnervation, detect absence of conduction during follow-up, or guide rehabilitation timing. As stated in the manuscript, the system was evaluated only on a healthy subject, and future work will calibrate these thresholds in nerve injury models. Although the present system is not a clinical diagnostic tool, its architecture suggests potential clinical benefits. The microstructured conduit may reduce axonal misrouting and neuroma formation by providing guided fascicular pathways. The portable monitoring system, with its simplified three-level classification (‘HEALTHY’, ‘WEAK’, ‘NO SIGNAL’), could support early detection of reinnervation, rapid screening during follow-up, and use in low-resource environments where full neurophysiological equipment is unavailable.

### 4.3. Limitations and Future Directions

Several limitations must be acknowledged. First, the conduits were fabricated exclusively for geometric and mechanical validation; no in vitro or in vivo biological testing was performed. Second, the monitoring system was evaluated only on a healthy subject under controlled stimulation conditions, which do not replicate the low-amplitude, variable signals expected during active nerve regeneration. Third, the current system does not include a synchronized stimulation channel, preventing the extraction of latency-based nerve conduction velocity (NCV), a key diagnostic parameter in clinical neurophysiology [17,18,19].

This study has several limitations. As stated in the manuscript, the conduits were fabricated only for geometric and mechanical validation, and no in vitro or in vivo biological testing was performed. PLA and standard SLA resins lack the flexibility required for implantable nerve conduits. The monitoring system was evaluated only on a healthy subject, and the FEA model used simplified loading conditions. Future work will incorporate flexible biocompatible materials, cytocompatibility assays, degradation studies, and validation in nerve injury models.

The monitoring system was evaluated only on a single healthy volunteer to verify the functionality of the acquisition chain. Future work will include testing on a larger cohort of volunteers to assess signal reproducibility and inter-subject variability, followed by validation in nerve injury models.

## 5. Conclusions

This study presents a design-stage engineering feasibility assessment of an integrated platform consisting of a microstructured 3D-printed nerve guidance conduit and a portable neuromuscular monitoring device. The fabrication results demonstrate that additive manufacturing can reliably reproduce internal microchannel architectures at both large-scale and miniaturized dimensions, supporting the suitability of 3D printing for rapid prototyping and structural optimization. The simplified finite element analysis confirmed that the conduit maintains mechanical stability under physiological pressure ranges, although the extremely small displacement values reflect the limitations of the pressure-only loading model and the absence of soft tissue interactions.

The portable monitoring system successfully acquired stable surface EMG signals during controlled stimulation, validating the correct operation of the amplification, filtering, and acquisition chain. The functional test performed on a single healthy volunteer served exclusively to confirm system operation and does not represent a physiological or clinical evaluation.

Overall, the results establish the structural and functional foundations required for future development of an integrated conduit-monitoring platform. The present work is intentionally limited to geometric, mechanical, and electronic validation. Future research will focus on material biocompatibility, in vitro characterization, expanded signal acquisition testing, and in vivo evaluation to further advance the system toward preclinical applicability.

## Figures and Tables

**Figure 1:**
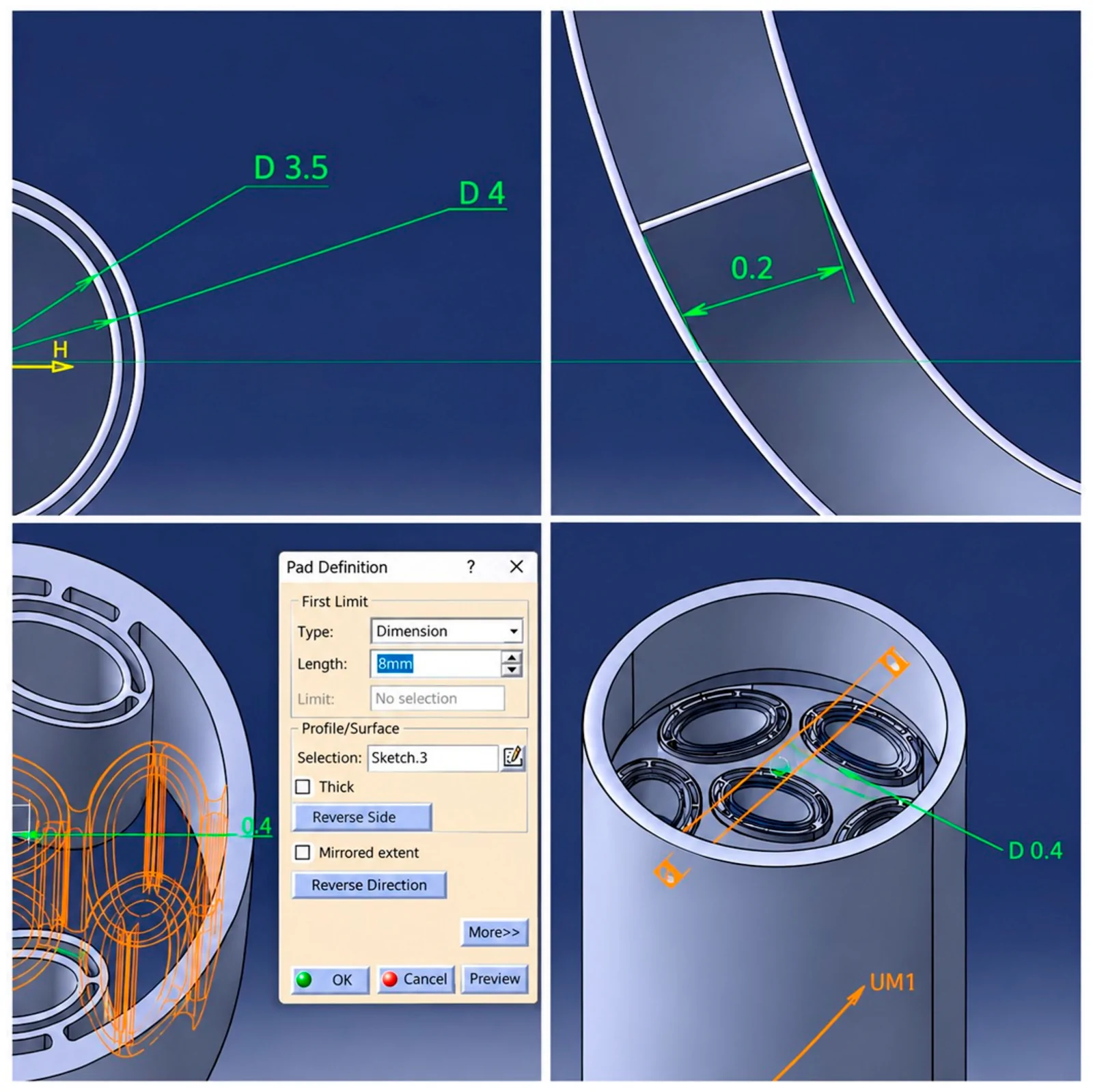
CAD design projections of Conduit A (6 fascicles). The conduit features an inner diameter of 3.5 mm, an outer diameter of 4 mm, an 8 mm active microchannel segment, 1 mm proximal and distal fixation ends, 0.4 mm surgical suture holes, and internal fascicular structures with a 0.2 mm wall thickness surrounding the central neurotrophic factor channel.

**Figure 2:**
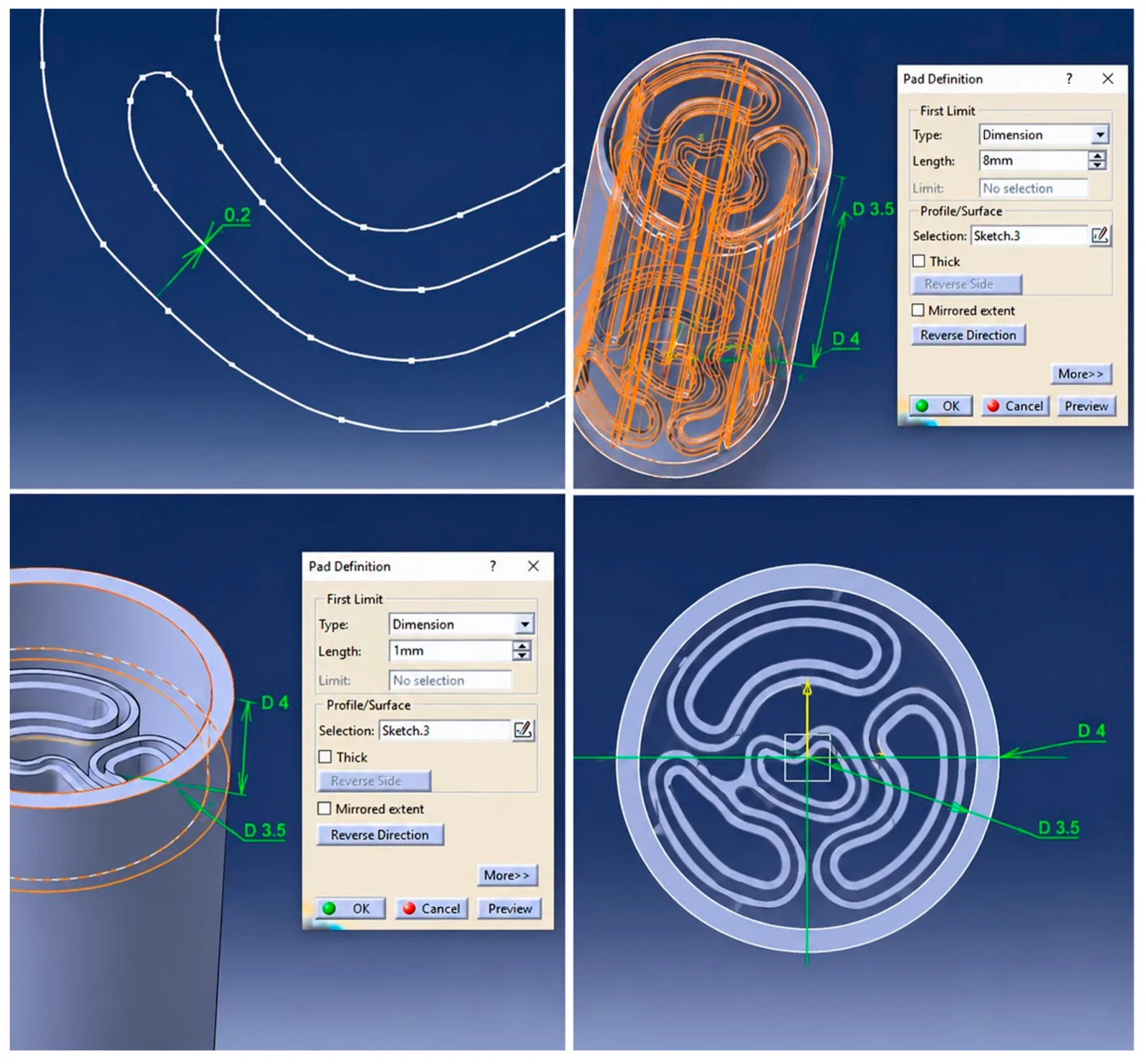
CAD design projections of Conduit B (4 fascicles). The conduit incorporates a 3.5 mm inner diameter, 4 mm outer diameter, an 8 mm active guidance segment, 1 mm proximal and distal fixation regions, 0.4 mm surgical suture holes, and internal fascicular structures with 0.2 mm wall thickness designed to support axonal guidance and future neurotrophic factor integration.

**Figure 3:**
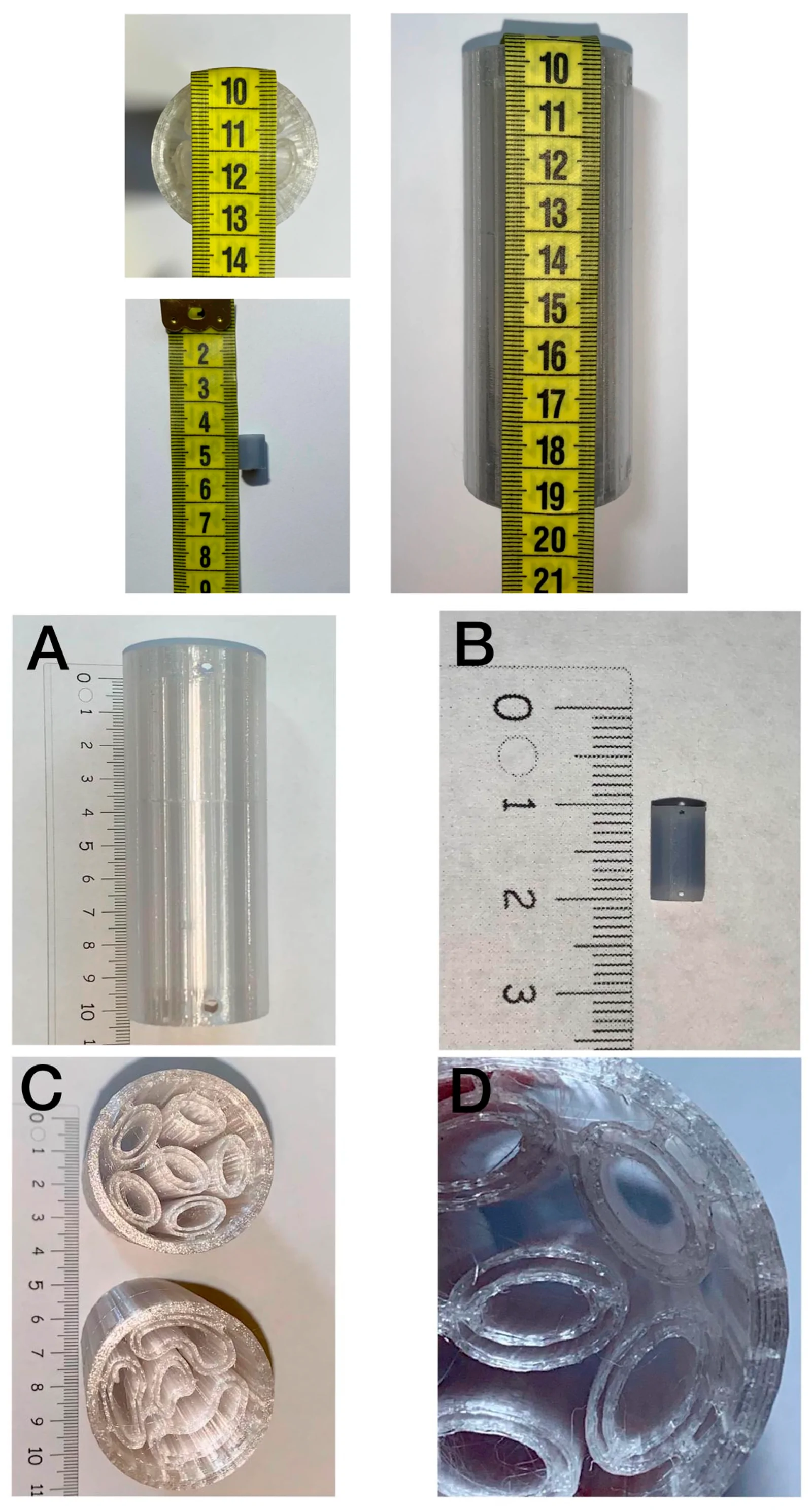
3D-printed nerve guidance conduit prototypes and structural features. (**A**) Longitudinal view of the large-scale FDM prototype (10 cm length). (**B**) Miniaturized SLA prototype fabricated at the target implantation dimensions (10 mm total length). (**C**) Cross-sectional views of the large-scale FDM prototypes illustrating the internal microchannel architectures of Conduit A and Conduit B. (**D**) Magnified view of the conduit cross-section demonstrating the open longitudinal fascicular microchannels and the central channel intended for future neurotrophic factor delivery.

**Figure 4:**
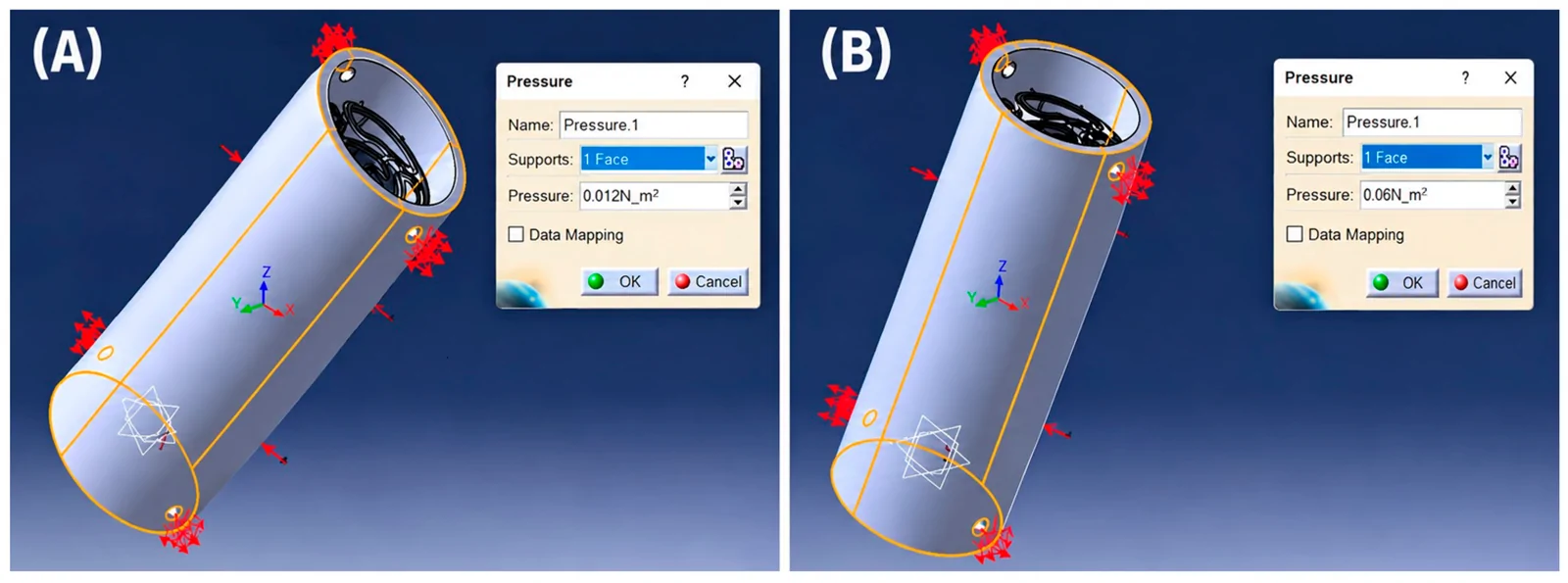
Application of distributed pressure load. (**A**) 1000 Pa (0.012 N/m^2^ distributed force) on the outer conduit surface. (**B**) 5000 Pa (0.06 N/m^2^ distributed force) on the outer conduit surface.

**Figure 5:**
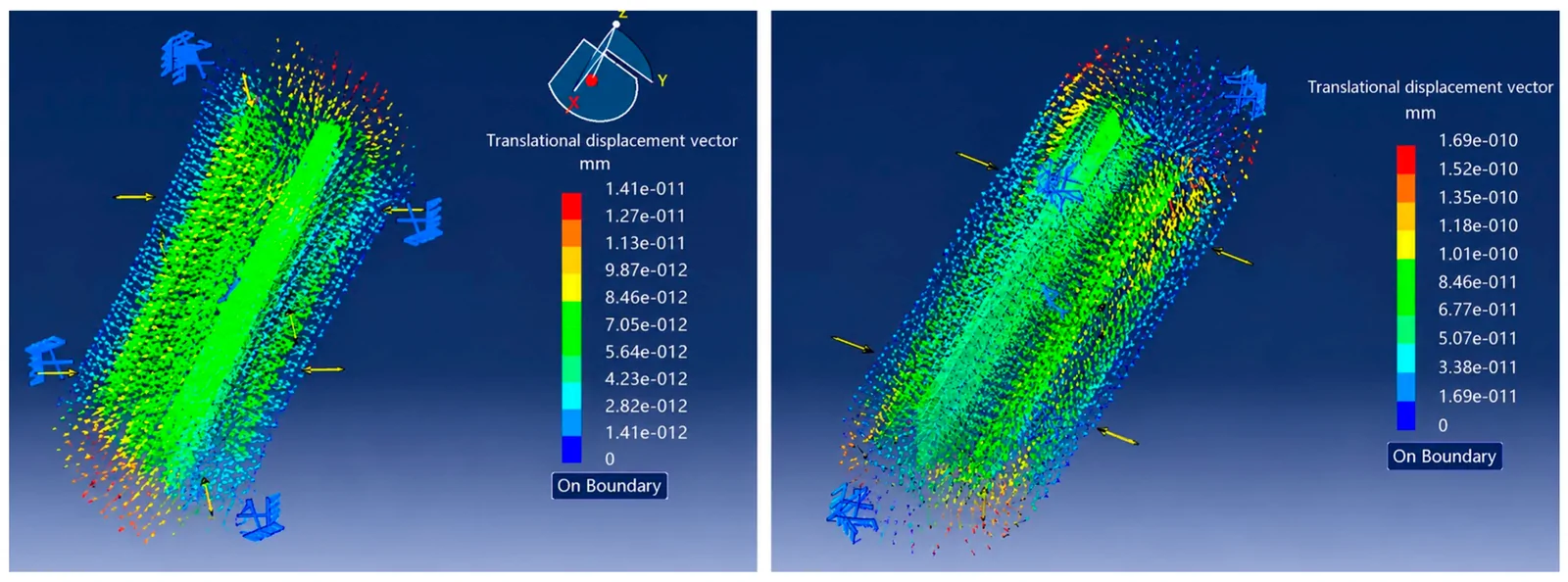
Displacement field maps.

**Figure 6:**
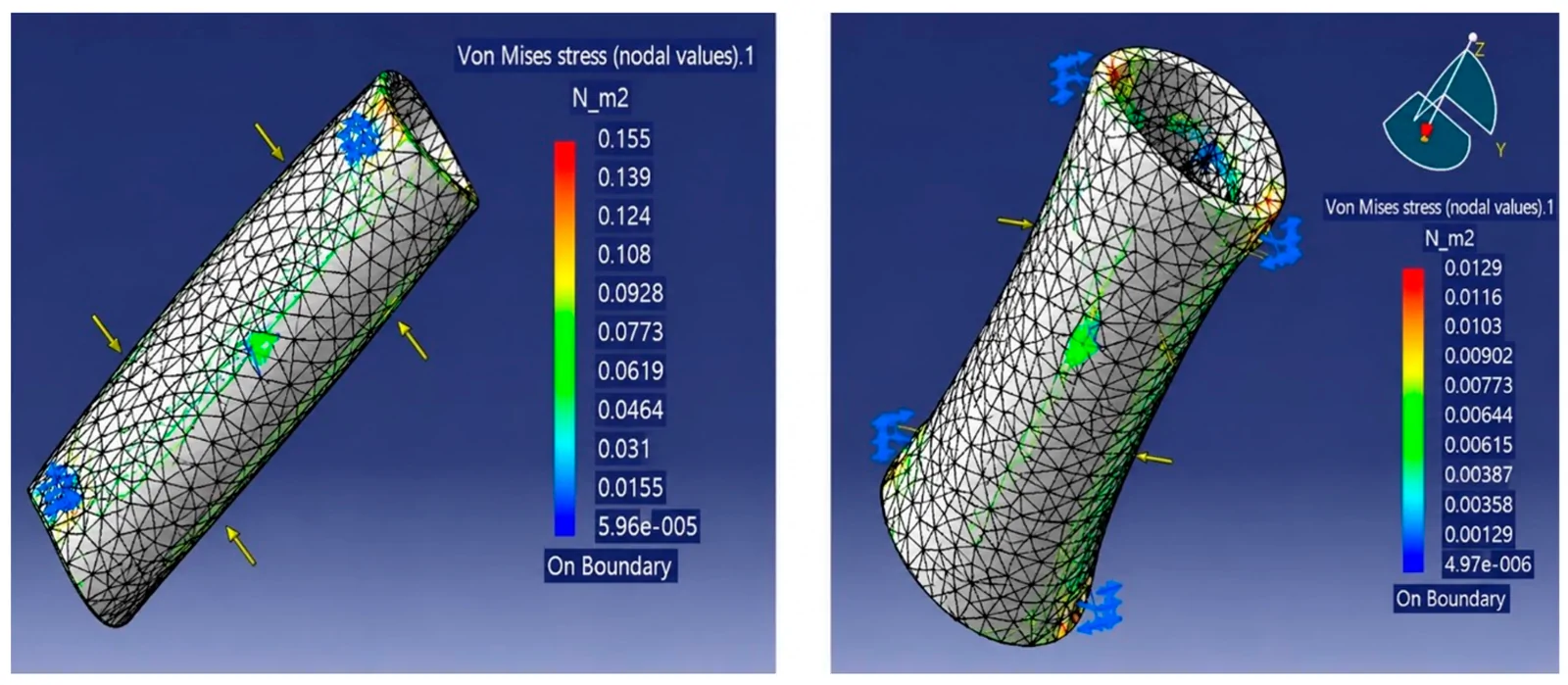
Von Mises stress distribution.

**Figure 7:**
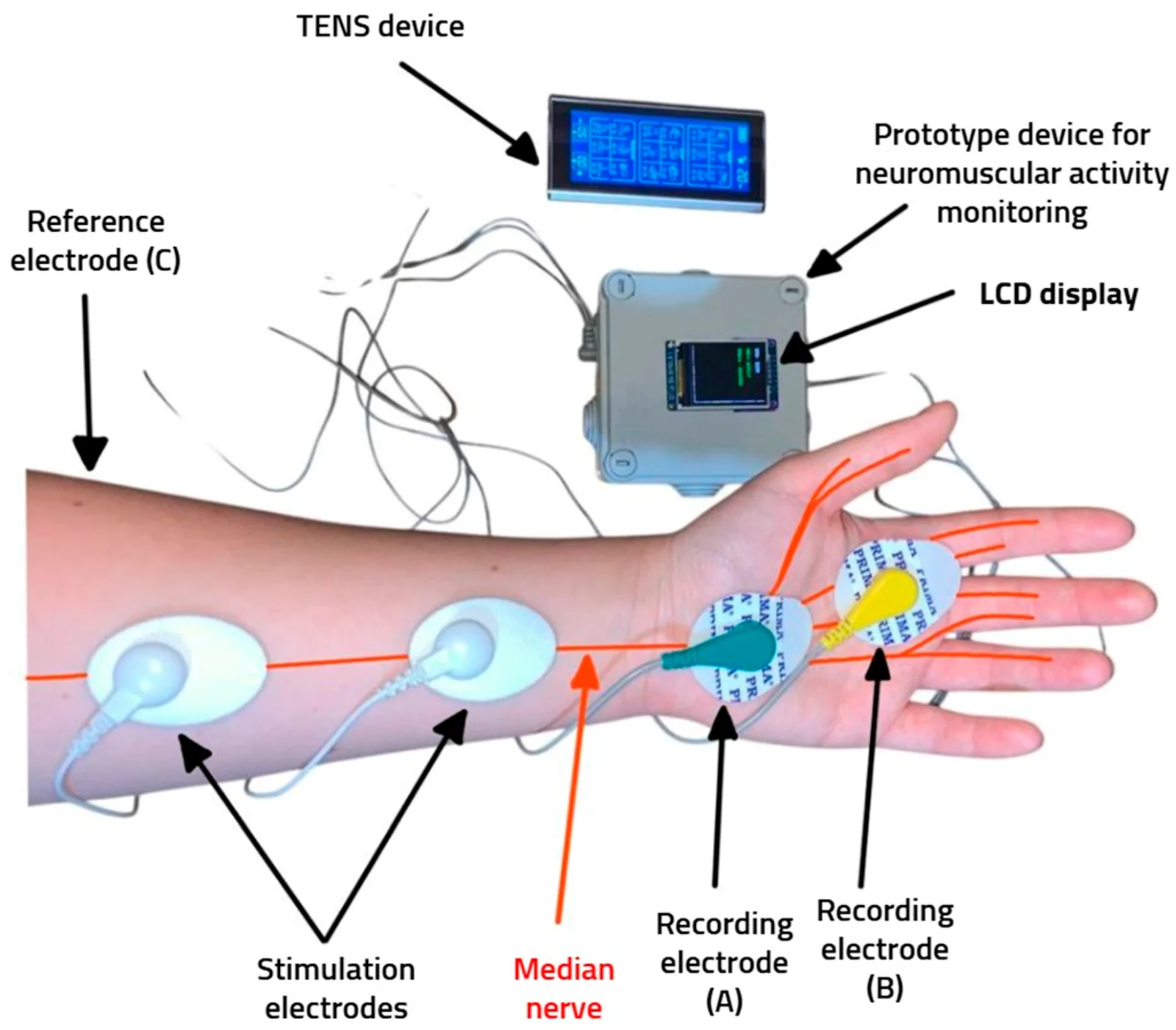
Experimental setup for neuromuscular activity testing.

## Data Availability

The original contributions presented in this study are included in the article. Further inquiries can be directed to the corresponding author.

## Abbreviations

The following abbreviations are used in this manuscript:

bFGF	Basic fibroblast growth factor
CMAP	Compound muscle action potential
EMG	Electromyography
FDM	Fused deposition modeling
FEA	Finite element analysis
NCS	Nerve conduction studies
NCV	Nerve conduction velocity
NGF	Nerve growth factor
PLA	Polylactic acid
SLA	Stereolithography
SNAP	Sensory nerve action potential
VDC	Volts direct current
